# The Book World of Medicine and Science

**Published:** 1905-04-29

**Authors:** 


### The Book World of Medicine and Science.

The Medical Annual. (Bristol: John Wright and Co. 1905. Price 7s. 6d.)

The "Medical Annual" answers a very useful purpose in bringing before the profession in a single volume and in concise form the chief new work which has been done in the different branches of medicine and surgery during the preceeding year. In reading the articles on the various subjects it must, of course, be remembered that most of the work which is recorded and the theories which are expounded are new and as it were *sub judice*, and therefore it must not be taken for granted that they are in all cases those to be accepted finally by the medical profession. During the past year the literature concerning head injuries has been much enriched by the lectures given by Rawlings and Crisp English before the College of Surgeons, and brief *résumés* of their papers have been included. Rawlings describes minutely the mechanics of fractures of the skull, and points out that most basic fractures result from forces applied directly to the basic level, and that these factures tend to travel across the base picking out the weakest spots. He considers as untenable the bursting and compression theories and the theory of fracture by contre-coup. Crisp English has carefully analysed the after effects of 300 cases of head injuries. He draws attention to the fact that the results are more serious than is generally supposed, and urges the necessity of more prolonged rest and more frequent trephinings in fractures of the vault without apparent depression. Under cleft palate Brophy's new operation for this malformation is described. In this operation the two palate and maxillary bones are drawn together by means of wire sutures. Hurry Fenwick, in his article on the surgical diseases of the kidney, describes and criticises the method of intravesical separation of the urine to which Bickersteth first drew the attention of the profession in April.

A most pleasing and useful addition to the volume is the incorporation of a number of stereoscopic plates which can be examined by means of a small hand stereoscope which is provided at an extra charge of 1s. 6d.

The Principles and Practice of Asepsis. By A. S. Vallack, M.B., M.Ch. (London: Baillière, Tindall and Cox. 1905. Pp. 105. Price 2s. 6d. net.)

The aim of the author of this volume has been "to describe a practical and efficient system of asepsis" and "to absolutely eliminate padding of any kind whatsoever." Dr. Vallack has succeeded in carrying out this aim, and has produced a carefully-written and well-thought-out book on asepsis. He begins by showing the necessity of seeing that the tissue resistance is in no way lowered, for "it is the tissues and not the surgeon who achieve the process of healing." Special stress is laid on the need of keeping the hands free, at all times, from septic matter. Gloves should be worn by everyone concerned in an operation, if it be a septic one, for when hands are rendered septic it is impossible to tell when they will become free again from infection. The instructions for preparing the patient, sterilising the hands, instruments, dressings, etc., though given in short compass are ample and efficient. The book, which closes with the treatment of septic and accidental wounds, is certainly well worthy of the surgeon's notice.

Surface Anatomy. By T. G. Moorhead. (London: Baillière, Tindall and Cox. 1905. Price 4s. 6d.)

As mentioned in the preface, the facts in "Surface Anatomy" can be found in standard works on anatomy and surgery. Mr. Moorhead has collected these facts and arranged them clearly and systematically, thus saving the medical student and practitioner much time. A diagram showing the relation of the superficial veins at the bend of the elbow might have been included, and some mention of the lymphatic system would have added to the value of the book. The book is well bound and excellently printed; the diagrams are good, although some of the numbers in red type are indistinct.

Clinical Lectures on Appendicitis, Radical Cure of Inguinal Hernia, and Perforating Gastric Ulcer. By G. R. Turner, F.R.C.S. (London: Baillière, Tindall and Cox. Pp. 136. 1905. Price 5s. net.)

This book consists of six lectures, two on operations for appendicitis, two on radical cure of inguinal hernia, and two on the treatment of perforated gastric ulcer. In St. George's Hospital Mr. Turner has operated on 140 cases of appendicitis with a mortality of 7·1 per cent., and he insists on the advisability of early surgical interference, to which he attributes the low mortality. To use his own words, "If there is going to be any doubt as to what the exact condition of the patient is, and whether he is likely or not to get better, then I maintain and hold very strongly that the safest thing to do is to operate." In the 140 cases 101 were acute, and of these 10 died (one, however, from chloroform), making a mortality of 9·9 per cent. In the lectures on hernia the author describes in detail the complete Bassini operation, giving his modification of it. Interesting figures and particulars regarding cases, with the results of operative treatment, are also given. In the last two lectures nine consecutive cases of perforated gastric ulcer are dealt with. Mr. Turner's teaching as to treatment of this condition is decisive. It is—Drain freely the flanks and Douglas' pouch as well as neighbourhood of ulcer, and irrigate the peritoneal cavity rather than use sponges or mops. The supra-pubic incision should rarely be omitted. The lectures contain much useful information.

Education through the Imagination. By Margaret McMillan. (London: Swan Sonnenschein and Co., Ltd. Pp. 196. Price 3s. 6d.)

Those interested in and having charge of the education of young children should not fail to read this book. Miss McMillan contends that the child-mind develops through the activity of the imagination, and that when this is suppressed, all the faculties are stunted. The book is well-written, and provides teachers with material for thoughtful consideration.

### BOOKS RECEIVED.

Baillière, Tindall and Co.

